# Reconstructing the Effectiveness of Policy Measures to Avoid Next-Wave COVID-19 Infections and Deaths Using a Dynamic Simulation Model: Implications for Health Technology Assessment

**DOI:** 10.3389/fmedt.2021.666581

**Published:** 2022-01-26

**Authors:** Arnold Hagens, Kathya Cordova-Pozo, Maarten Postma, Jan Wilschut, Lorenzo Zino, Jurjen van der Schans

**Affiliations:** ^1^Department of Health Sciences, University of Groningen (RUG), University Medical Center Groningen, Groningen, Netherlands; ^2^Institute for Management Research, Radboud University, Nijmegen, Netherlands; ^3^Department of Economics, Econometrics and Finance, Faculty of Economics and Business, University of Groningen, Groningen, Netherlands; ^4^Center of Excellence in Higher Education for Pharmaceutical Care Innovation, Universitas Padjadjaran, Bandung, Indonesia; ^5^Department of Pharmacology & Therapy, Universitas Airlangga, Surabaya, Indonesia; ^6^Department of Medical Microbiology, University Medical Center Groningen, University of Groningen, Groningen, Netherlands; ^7^Faculty of Science and Engineering, University of Groningen, Groningen, Netherlands

**Keywords:** SIRD model, simulation, COVID-19 scenarios, differential measures, vaccination, intergenerational contacts

## Abstract

**Objective:**

The goal of this study was to dynamically model next-wave scenarios to observe the impact of different lockdown measures on the infection rates (IR) and mortality for two different prototype countries, mimicking the 1st year of the COVID-19 pandemic in Europe.

**Methods:**

A dynamic simulation SIRD model was designed to assess the effectiveness of policy measures on four next-wave scenarios, each preceded by two different lockdowns. The four scenarios were (1) no-measures, (2) uniform measures, (3) differential measures based on isolating > 60 years of age group, and (4) differential measures with additional contact reduction measures for the 20–60 years of age group. The dynamic simulation model was prepared for two prototype European countries, Northwestern (NW) and Southern (S) country. Both prototype countries were characterized based on age composition and contact matrix.

**Results:**

The results show that the outcomes of the next-wave scenarios depend on number of infections of previous lockdowns. All scenarios reduce the incremental deaths compared with a no-measures scenario. Differential measures show lower number of deaths despite an increase of infections. Additionally, prototype S shows overall more deaths compared with prototype NW due to a higher share of older citizens.

**Conclusion:**

This study shows that differential measures are a worthwhile option for controlling the COVID-19 epidemic. This may also be the case in situations where relevant parts of the population have taken up vaccination. Additionally, the effectiveness of interventions strongly depends on the number of previously infected individuals. The results of this study may be useful when planning and forecasting the impact of non-pharmacological interventions and vaccination campaigns.

## Introduction

The ongoing COVID-19 pandemic caused by the severe acute respiratory syndrome coronavirus 2 (SARS-CoV-2) has caused over 100 million infections and more than 2 million deaths worldwide by the start of 2021 (1). COVID-19 transmits by small droplets which contain the virus that are breathed in or land on surfaces which then reach the respiratory system through contact (2). At the beginning of the COVID-19 epidemic, the spread of the virus was fast with an initial reproduction number (R0) in the naive population varying between 2 and 4 causing the infection to spread rapidly across the globe (3). The effective reproduction number (R_t_) has then started decreasing, following the implementation of non-pharmaceutical intervention policies and social measures to prevent contagions (4, 5). Starting from March 2020, many European countries have enacted lockdown measures to control the viral transmission, which entailed enforcing staying or working at home, closing schools, or implementing travel bans (6, 7). By the end of July 2020, most of the countries in Europe appeared to have the virus under control. Hence, lockdown measures were relaxed toward reducing their social and economic negative impact, despite the World Health Organization (WHO) warned for the risk of resurgent waves of infections (8). Indeed, by the end of 2020, next waves did occur in many countries across the globe. With current vaccines' rollout and the fear related to the emergence of new strains, potentially resistant to vaccines, herd immunity is still far from being reached in European countries. Hence, it is crucial to understand the dynamics of the pandemic and the effectiveness of policy measures to identify the most effective non-pharmacological measures and for optimal planning for the next steps of vaccination campaigns and possible impacts of new COVID-19 strains.

We based our study on the following observations. Consistently across countries, between 80 and 95% of reported deaths occurred among adults aged 60 or older (9–11), which are the age cohort more at risk of developing severe consequences. Aiming at high-risk groups in specific interventions has also been simulated by Akamatsu et al. who also mentions this in a proposed strategy (12). Yet, major differences exist between countries, concerning the rates of infections, hospitalizations, and death. We argue that some of these differences may be traced back to the different interventions that were implemented across the countries (13). Additionally, underlying sociodemographic data show key differences between European countries. Importantly, through the analysis of contact patterns, it emerged that individuals in the 60 years or older age category living in Southern European countries have about two times as much social interactions compared with the same age cohort in many Northwest European countries (14). These differences in social activity, together with different age distributions and intergenerational contact patterns, have been recognized to be potentially a key factor in determining the higher rates of COVID-19-related mortality in countries such as Italy as compared to, for example, the Netherlands (15).

Since 25% of the European population is in the high-risk age cohort (> 60 years old) (16), it is of paramount importance to carefully consider the balance between protection, transmissibility, and consequences of a viral infection in different age groups when planning the implementation and release of lockdown measures, with a look to their social and economic impact. Indeed, lockdown measures can consist of different actions such as staying or working at home, wearing face masks, and enacting social distancing, often applicable generically to all age groups and implemented uniformly across them. However, considering the different risks of viral infection and the related consequences of COVID-19, in different age groups, the assessment of differential measures among age groups needs to be addressed. This is especially important when considering the economic consequences of the ongoing pandemic. In fact, the costs related to the epidemic are anticipated to affect the real GDP growth in the European Union between −10.8 and 3.4% in 2020 (17). Such an estimation would possibly be worse if extreme and long-lasting lockdown measures need to be implemented, but it can potentially be mitigated with a more differentiated approach among age groups to return to an economically productive life, which could also to reduce the sociopsychological consequences for younger age groups (18, 19).

The objective of this study is to retrospectively evaluate the effectiveness of the various scenarios to reduce or avoid a next wave of COVID-19 in terms of averted infections, and deaths, taking specific characteristics of age groups and their contact patterns explicitly into account. Specifically, we aim at comparing an age-based differential lockdown approach with rather generic or uniform approaches. In view of the ongoing vaccination campaign, our analyses are performed in the context of an early-stage immunization program that will stepwise expand from high-risk groups to the entire population, during 2021. From our retrospective analysis, we derive important insights for Health Technology Assessment into the effectiveness of different lockdown measures and next-wave behavior, which may be useful for informing and modeling vaccination and other intervention strategies while considering the impact of previous waves on the outcome of each intervention.

## Materials and Methods

**Figure 1:**
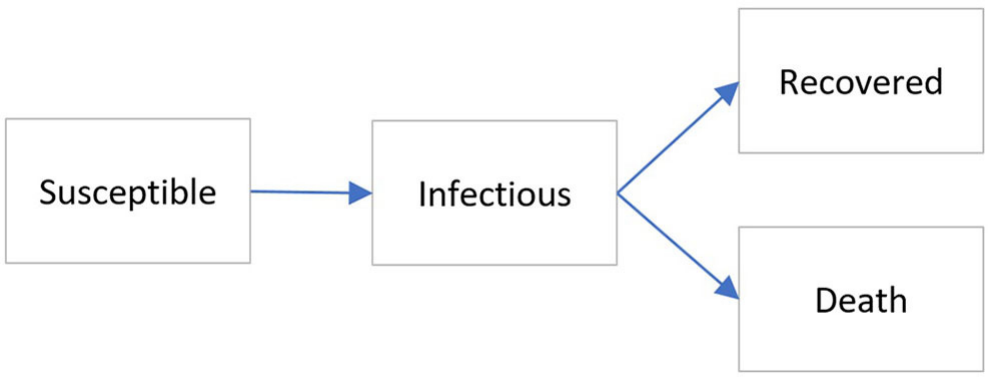
Simplified representation of the used SIRD model.

We developed a dynamic simulation 4 age groups' SIRD (susceptible, infectious, recovered, and dead) model (Figure 1) to evaluate the actual effectiveness of different lockdown measures by considering the impact of measures implemented in the previous wave(s). In this model, the S compartment reflects the number of susceptible persons, the I compartment the number of infectious, the R compartment the number of persons recovered and immune, and the D compartment the number of deaths. The SIRD model was chosen as it suits our objective of evaluating effectiveness while preventing unnecessary complexity. For this study, we define lockdown measures as the set of measures with the objective to reduce movement of people and contacts. The SIRD model was simulated using the Runge-Kutta method to approximate the solutions of the differential equations (Appendix B). The contact matrix, R_0_, and the infectious period are used for simulating the new infections and the recovery rate vector for simulating distributions of recovered and deaths across the age groups. All the parameter used are reported in Table 1.

**Table 1:** Summary of parameters used.

**Variable**	**NW**	**S**	**Reference/ assumption**
**Country characteristics**			
Prototype population size	10,000,000	10,000,000	Fictitious
0–19 years	22.3%	17.8%	(20, 21)
20–39 years	24.3%	21.8%	“
40–59 years	28.2%	30.8%	“
≥ 60 years	25.2%	29.7%	“
Contact patterns	Low	High	(14), Appendix A
Case fatality rate (CFR)	Estimated		Appendix C
0–19 years	0.000%		
20–39 years	0.003%		
40–59 years	0.040%		
≥ 60 years	7.700%		
Infectious period (days)	7		(22)
R0	2.8		(4)
**Days**			
First-wave policy starts at day	51		(9, 23–27)
First-wave policy duration (days)	125		^*^
Second-wave policy starts	176		Day that wave ended
Effectiveness of the policy (range)	0–100%		

* *On average, the measures in place lasted 125 days*.

### Prototype Countries

To evaluate the effectiveness of the scenarios in two different settings, prototype countries were created. A set of population characteristics from the Netherlands and Italy were used to construct two prototype countries, potentially reflecting more general situations in North-West (NW) and Southern (S) Europe, respectively. Effects of similar policies in these two different settings were analyzed for a fictitious population of 10,000,000 inhabitants. The prototype countries were different concerning age distributions, with the NW having a larger proportion of the population in the age group from 0 to 39 years and a smaller proportion in the ≥ 40 years of age group compared with S (Table 1). Four age groups were defined in each prototype: 0–20, 20–40, 40–60, and older than 60 years. In addition, the prototypes differed in terms of intergenerational contact patterns. The contact patterns of a country are reflected in the social contact matrices (14). These matrices report the average number of daily contacts of individuals within their own age group and between different age groups for different locations, including households, schools, workplaces, and other locations. The contact matrix is the core of the simulation of the spread of respiratory infections. The base contact matrix for each country was constructed with real-world data and then aggregated in four age groups for this study (28). We observe that S has a higher number of contacts among older adults compared with NW (19). The base contact matrix was included in the construction of every scenario, and additional matrices were derived for the *differential measures* scenarios according to the lockdown measures specified in Table 2 (see also Appendix A). It is worth noticing that our prototypes, despite being shaped on specific countries, are representative of groups of countries with similar sociodemographic characteristics. For example, Belgium and United Kingdom have a similar share of ≥ 60-year-old population as the Netherlands, and Bulgaria and Greece have a similar share of ≥ 60-year-old population as Italy (28).

**Table 2:** Simulated policy measures for the scenarios.

**Scenario**	**Home**	**School**	**Work**	**Other**
No-measures	No limitations are enforced			
Uniform measures	57% uniform reduction of contacts (general measures such as distancing, face masks, etc.)			
Differential measures A	All home contacts normal	Only aged under 60 years have normal school contacts	Only 20–60 years have normal works contacts	All contacts in other locations are forbidden for all age groups
Differential measures B	All home contacts normal	Only aged 0–20 year have normal school contacts	Contacts for all aged over 20 are reduced by 70%	All contacts in other locations are forbidden for all age groups

### Parameters Used

The input parameters used in this study are summarized in Table 1. On average, the initial lockdown measures in the first wave started on day 51 (47–54), which is the average number of days between the day of the first positive case and the beginning of the lockdown measures in Italy and the Netherlands (9, 23–27). The duration of lockdown measures in the previous wave was estimated at ~125 days. European countries introduced lockdown measures during the month of March and started reducing them as of June 2020. However, some degrees of lockdown measures stayed in place in some countries and have been reinstalled or reinforced again in the end of 2020^1^. The case fatality rate (CFR) expresses the number of fatalities per confirmed (PCR-positive) case. For the model, all infections are assumed to have been detected and confirmed. The first wave was used as a base situation for the simulation of the various scenarios. The CFR for the different age groups in the two prototypes was estimated based on the number of reported deaths in the Netherlands, up to July 7, 2020 (29), the moment when there were few new daily and active cases and sufficient data were available to perform the analysis. Next, the number of infections according to the NW model (adjusted for the real population size) was used to calculate the CFR for each age group. When corrected for the additional reported infections, the number of total infections in the NW model also coincided with estimates of the Dutch blood bank (Sanquin) that 6.5% of the population had been infected (antibody study) by May 18, 2020 (30) (Appendix C). The used CFR is also similar to estimates for the USA by Levin et al. (31).

### Modeled Scenarios

**Figure 2:**
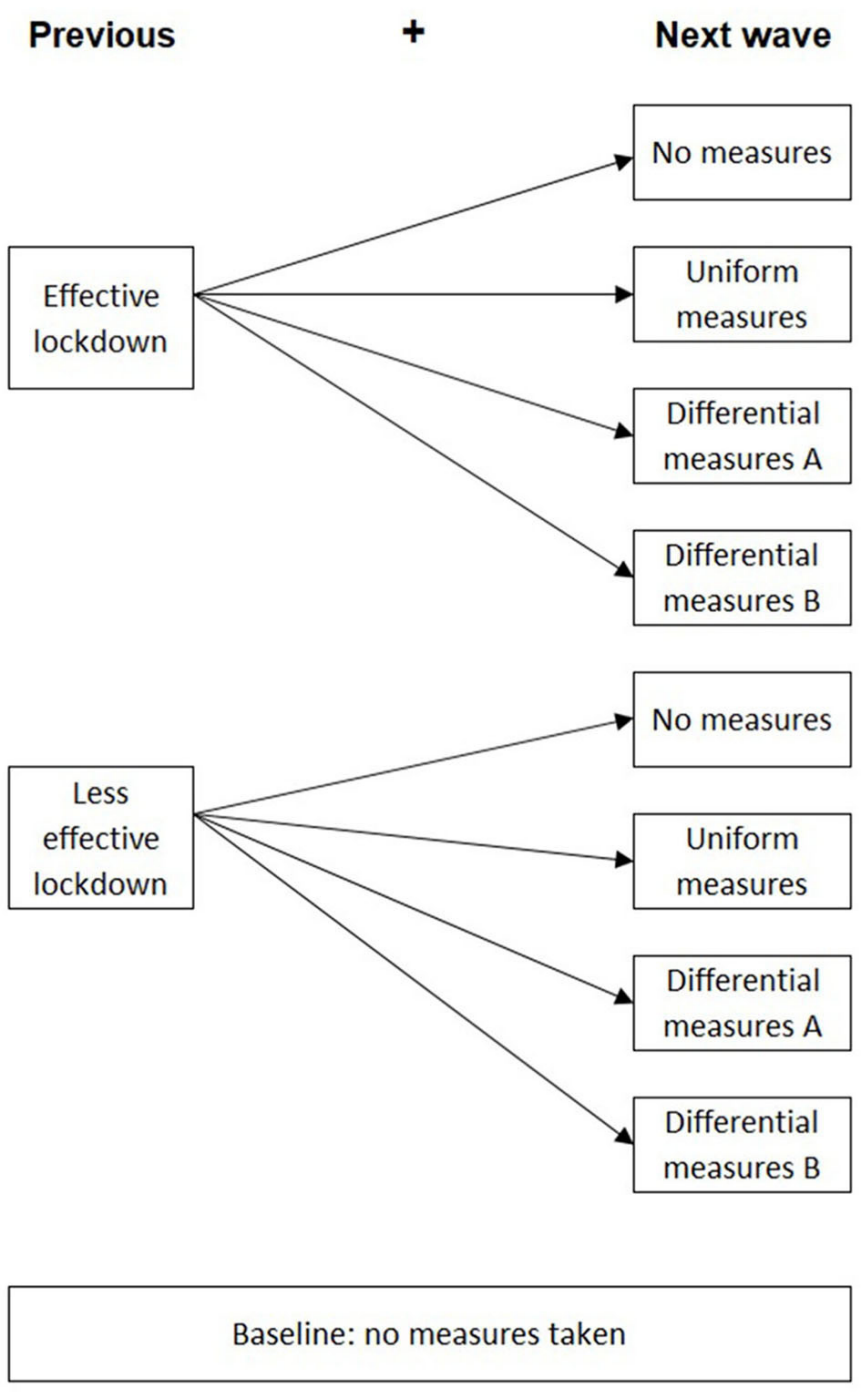
The eight simulation scenarios including the previous lockdowns and the next-wave scenarios.

After the first lockdown period of 125 days, eight next-wave scenarios were modeled to observe the incremental deaths and the immunity ratio (ImR) for the two different prototypes. The previous lockdown was differentiated between effective and less effective previous lockdowns, as described below (Figure 2). The next-wave scenarios included four plausible policy options for each next-wave lockdown, that is, (1) *no-measures*, (2) *uniform measures*, (3) *differential measures A*, and (4) *differential measures B*, which are detailed in section Next-Wave Scenarios. A time span of 1,000 days was used for the simulation to observe the number of daily infected people to decrease to near zero in all the scenarios.

### Previous-Wave Lockdowns

In our model, an effective previous lockdown assumed that actions taken to reduce the spread of the virus resulted in a reduction of R0 from 2.8 to <1, resulting in the epidemic to die out. For the effective previous lockdown, we set the effectiveness at 73% resulting in an R0 = 0.75. This effectiveness level is based on the situation in the Netherlands, Germany, and Italy, after implementation started of the lockdown measures. In fact, the estimated R_t_ values in these countries during the lockdown varied from 0.76 to 0.82 (32).

A less effective previous lockdown was defined as one in which the actions that intended to reduce the Rt did not manage to decrease it below 1. For example, Sweden had R_t_ ≥ 1 during the first-wave lockdown. For the simulation of a less effective lockdown, we used a resulting R0 = 1.2 that means an effectiveness of 57% ($\frac{\left(2.8-1.2\right)}{2.8}$). In this scenario, the virus is not under control and daily infections keep increasing until the number of susceptible persons is low enough for the epidemic to die out. The R_t_ in lockdown (for the different reference countries) were estimated with time-dependent estimation of daily new COVID-19 cases from ECDC data (1, 31).

Each of the described previous lockdowns formed the basis for the dynamic simulation of the next wave and the subsequent policy measures as described below.

### Next-Wave Scenarios

In the next-wave phase, four different scenarios of intervention policies were modeled. In the *no-measures* scenario, the virus is free to spread without the imposition of any restrictive measures. This scenario was chosen as a comparative scenario. However, it was assumed that as the epidemic advances, people's behavior would change due to visible infections and deaths around them, and that the R_0_ would no longer be 2.8, but rather 1.8. The assumption is supported by R_t_ collected between April 1, 2020 and April 1, 2021, which shows approximately only 1% of the reported R_t_ to be above 1.8 (Appendix E) that can be interpreted as that even the poorest set of measures reached an R_t_ of 1.8.

In the *uniform measures* scenario, the policy was assumed to be the same throughout the entire population. This strategy was implemented in many countries during the first wave, enforcing equal restrictions for the various age groups. A conservative effectiveness value was assumed for this scenario, equal to 57%, that is, equal to the less effective previous lockdown. Such a choice was motivated by empirical observations in Google Mobility data of a reduced adherence to strict measures comparing the first to second wave in various countries in Europe (33).

**Figure 3:**
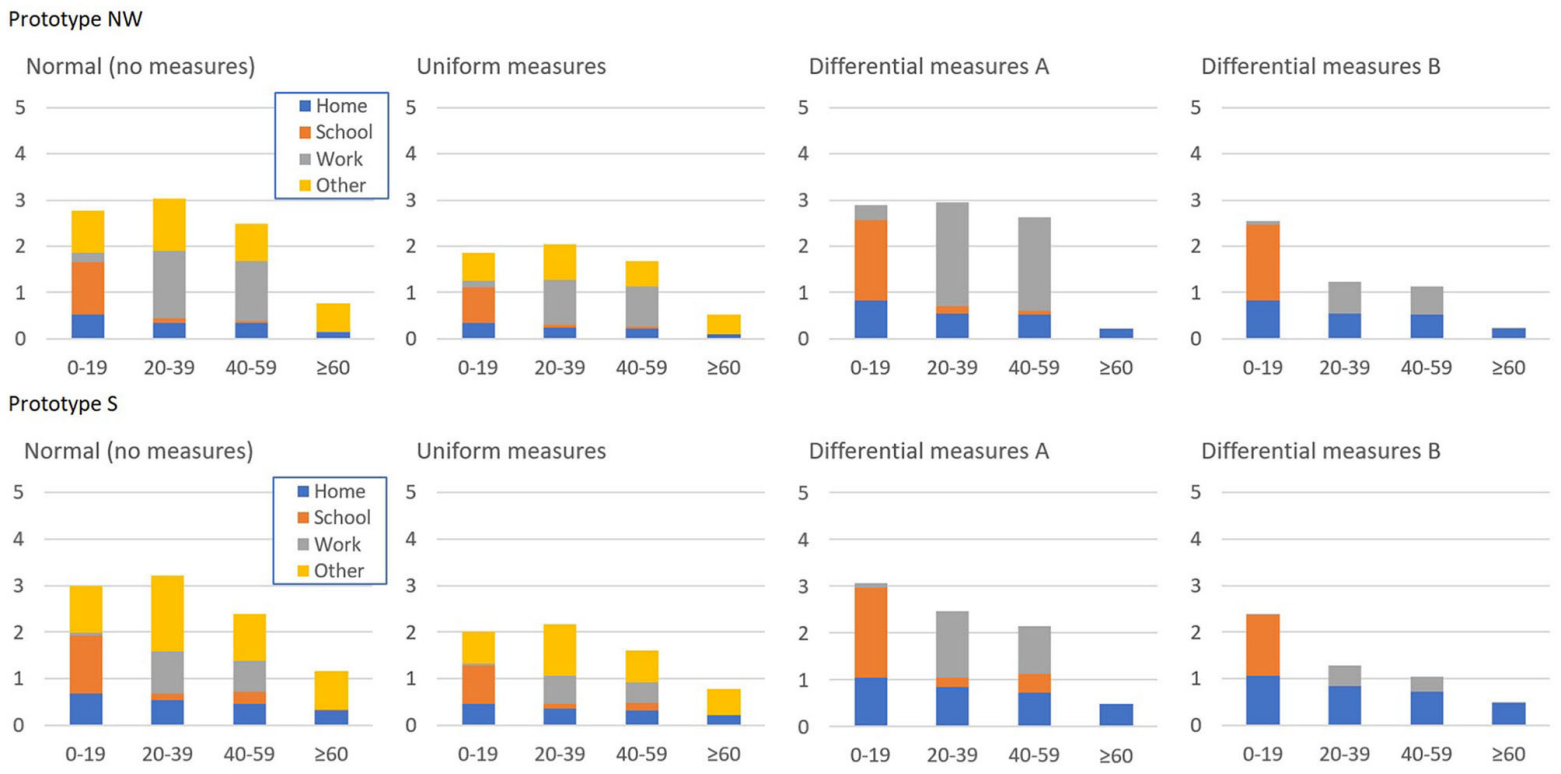
Per-person contacts per day in the next-wave scenarios for the two prototypes.

Finally, we considered two *differential measures* scenarios. In these scenarios, the imposed policy was differential in the sense that only specific contacts in the base-contact matrix of the two prototype countries were reduced. For this reduction, two dimensions were used: the age group and the location (home, school, work, other locations). The *differential measures A* scenario was designed with contacts at home continuing as normal, only the 0–60 age group continuing school contacts and only the 20–60 age group maintaining work contacts. Contacts in “other locations” would be forbidden for all age groups. In the *differential measures B* scenario, contacts at home could resume as normal, but only the 0–20 group could resume with school contacts and the ≥ 20 age group could maintain only 30% of work contacts using distancing, face masks and other general preventive options. Contacts in “other locations” would be forbidden for all age groups. The main objective of the *differential measures B* was to analyze the impact of reducing work-related contacts in addition to reducing school contacts by distancing the teachers and implementing home schooling (see Table 2 and Figure 3). For both *differential measures scenarios, it was assumed that no loss of adherence will occur as differential measures are less random, more specific, legitimate, and there are less economic reasons for non-adherence*. The contact matrices for both *differential measures* scenarios are shown in Appendix A.

### Data Analysis Procedure

For the 8 scenarios, the number of infections and deaths was simulated and logged by day. From these data, the situation was determined at the start of the next wave and at the end of the next wave. For the next wave scenarios, additionally incremental deaths per age group were registered. For the simulation and data analysis, Vensim 8.0.9 and Excel version 2109 were used.

## Results

Before we extensively present and discuss the results of our study, it is worth commenting on the status of the population at the start of the next-wave scenario. In fact, a key piece of information for the start of the next-wave scenarios concerns the number of immune persons at the end of the previous lockdown (Table 3). An effective previous lockdown is estimated to end with an ImR of 0.14, which is the ratio between immune persons and the total population. This ImR is significantly lower than the final ImR of 0.34 and 0.35 (prototype NW and S), respectively, at the end of the less effective previous lockdown. It should be noted that due to the uncertainty of the underlying parameters used, these ImRs are an approximation.

**Table 3:** Cumulative infections, deaths, and share of immune persons over the whole population, ImR of the two previous lockdown types at the start of the next wave scenarios.

**Lockdown type**	**Infections**	**Deaths**	**Immunity ratio**
**Prototype NW**			
Effective previous lockdown	1,407,900	6,236	0.14
Less effective previous lockdown	3,448,490	17,996	0.34
**Prototype S**			
Effective previous lockdown	1,422,960	12,814	0.14
Less effective previous lockdown	3,467,450	35,738	0.35

In Table 4, the results of the different simulation scenarios for the two prototype countries are presented. The table reports the incremental deaths and their share per age group of the next-wave scenarios, the end ImR, the increment of the ImR during the next wave, and the cumulative death toll of all waves. Appendix D shows the development in time of weekly infections and deaths.

**Table 4:** Effectiveness of the policy measures over four scenarios for two prototype countries showing the incremental deaths and their share per age group of the next-wave scenarios, the ImR, the increment in the ImR, and the cumulative deaths in all waves.

**Next-wave scenario**	**Incremental deaths in next-wave scenario**							**End state**		**Cumulative deaths all waves**
	**0–39**		**40–59**		**≥60**		**Total**	**Immunity ratio**	**Incremental immunity ratio**	
	**#**	**Share**	**#**	**Share**	**#**	**Share**				
**Prototype NW**										
**Effective previous lockdown** ^**a**^										
1. No-measures	35	0.1%	491	1.8%	26,376	98.0%	26,902	0.52	0.38	33,138
2. Uniform measures	0	0.2%	4	2.5%	150	97.3%	154	0.14	0.00	6,390
3. Differential measures A	37	1.2%	554	18.0%	2,491	80.8%	3,082	0.52	0.38	9,319
4. Differential measures B	4	0.7%	57	9.8%	524	89.5%	585	0.22	0.08	6,822
**Less effective previous lockdown** ^**a**^										
5. No-measures	3	0.1%	45	1.8%	2,489	98.1%	2,537	0.38	0.04	20,534
6. Uniform measures	0	0.1%	3	1.8%	175	98.0%	179	0.35	0.00	18,175
7. Differential measures A	6	1.1%	93	17.0%	445	81.9%	543	0.41	0.06	18,540
8. Differential measures B	0	0.2%	3	3.1%	80	96.7%	82	0.35	0.00	18,078
**Prototype S**										
**Effective previous lockdown** ^**a**^										
1. No-measures	32	0.1%	513	1.0%	50,270	98.9%	50,815	0.53	0.38	63,629
2. Uniform measures	0	0.1%	3	1.3%	215	98.6%	218	0.14	0.00	13,032
3. Differential measures A	22	0.2%	416	3.1%	13,186	96.8%	13,624	0.42	0.27	26,437
4. Differential measures B	0	0.1%	4	1.7%	209	98.2%	213	0.15	0.01	13,026
**Less effective previous lockdown** ^**a**^										
5. No-measures	3	0.1%	47	1.0%	4,712	99.0%	4,762	0.38	0.04	40,500
6. Uniform measures	0	0.1%	3	1.0%	329	98.9%	332	0.35	0.00	36,070
7. Differential measures A	1	0.1%	14	2.6%	508	97.2%	522	0.35	0.01	36,260
8. Differential measures B	0	0.1%	2	1.1%	199	98.8%	201	0.35	0.00	35,939

a *The Immunity ratio at the start of the next-wave scenarios was calculated at 0.14 (for NW and S) and 0.34 (NW) and 0.35 (S) for effective and less effective previous lockdowns for both prototypes*.

*Share: Share of the total new deaths within the next-wave scenario*.

*Incremental immunity ratio: Difference of ImR at start and after next-wave scenario*.

*Total population: 10 million*.

*End state: The situation after the next-wave scenario has plateaued (day 1,000)*.

Comparing the effective previous lockdown followed by the *no-measures* scenario (scenario 1) with the less effective previous lockdown followed by the *no-measures* scenario (scenario 5) showed less incremental deaths for the less effective lockdowns than in the effective lockdowns, 2,537 and 26,902, respectively, for prototype NW. This is a consequence of the effective previous lockdown causing a lower ImR (0.14), compared with the ImR of 0.34 after the less effective previous lockdown, which implies a higher number of susceptible persons in the next-wave *no-measures* scenario. This effect also results in fewer cumulative deaths in the *no-measures* scenario preceded by the less effective previous lockdown than in the *no-measures* scenario preceded by an effective previous lockdown scenario (20,534 and 33,138, respectively). Additionally, the share of incremental deaths of persons older than 60 was 98.0 and 98.1% for the effective and less effective previous lockdown, respectively.

The *uniform measures* scenarios (scenarios 2 and 6) showed fewer and indeed low incremental deaths compared with the *no-measures* scenario, both in the case of an effective and a less effective previous lockdown (154 and 179, respectively). The ImR increase stayed below 0.005. An effective previous lockdown followed by the *differential measures A* scenario (scenario 3) showed more total incremental deaths compared with *uniform measures* (3,082 and 154, respectively) and increased the ImR with 0.38. Scenario 7, a less effective previous lockdown followed by *differential measures A*, showed only little more incremental deaths than in the *no-measures* scenario (543 and 179 for NW, respectively) and an increase of the ImR with 0.06. The difference between the effective and less effective lockdown is again caused by the higher ImR at the beginning of the next wave, causing a lower increase of the ImR and fewer incremental deaths. Additionally, the share of incremental deaths for the ≥ 60-year-old group for the *differential measures A* scenario is reduced to 80.8 and 81.9% for the effective and less effective previous lockdown, respectively. This is achieved by selectively allowing certain locations and restricting contacts by age group especially the ≥ 60 group (see Figure 1 and Table 2). An effective previous lockdown followed by the *differential measures B* scenario (scenario 4) showed more, but still rather low, total incremental deaths compared with *uniform measures* for NW (585 and 154, respectively). For the less effective previous lockdown followed by *differential measures B*, this was 82 and 179, respectively.

The results also showed that the incremental deaths are higher or similar in prototype country S compared with NW. A small exception relates to scenario 4 for NW where the incremental deaths are 585 and higher than in S (213). A closer look into the model's parameters showed this was to a large extent due to the high population share under 20 years in prototype NW with a high number of contacts with other age groups. Additionally, the *differential measures A* scenario in prototype country S was not as effective in isolating and reducing the share of deaths of people older than 60 as in prototype NW (80.8% and 96.8% for NW and S, respectively).

## Discussion

This study quantifies the effectiveness of policy measures to avoid next-wave COVID-19 infections and deaths in two prototype countries with differences in age distribution and intergenerational contact matrices, using a dynamic compartmental SIRD model. Our results show that restrictive lockdown measures are effective, and that the specific nature of these measures is important during a subsequent wave of COVID-19 viral transmission. However, the effectiveness depends on the initial epidemiological situation and of the next-wave behavior; therefore, control measures need to be adjusted for this. Additionally, we showed that countries with a higher share of persons older than 60 need to consider different or stricter measures to reduce infections and deaths in that age group.

In the model that we used to describe the course and the impacts of the epidemic waves, we made several assumptions. First, the CFR was estimated using simulated cases, in line with the numbers found in serological antibody studies (30), and the reported number of deaths from the Netherlands (1). Appropriate serological studies for Italy were not available, and therefore, the same CFRs were used for each prototype. Nevertheless, the CFR is likely to be an overestimate of the real number of infections (reported and unreported) since not all positive cases were detected. A lower CFR would result in less deaths in the model. As age trends are expected to stay the same, a lower overall CFR would affect the mortality in each age group; especially, the population older than 60 years would be affected. Second, an R_0_ of 1.8 is used in the situation that a population is aware of the virus and its effects, but without any forced measures. Since people will probably act differently and be more careful when they see infections, hospitalizations, and deaths in their immediate surroundings, we assume the R_0_ may be lower than the R_0_ of 2.8, even in a situation without any measures enforced by public authorities (34). Finally, to reflect the effectiveness of a less effective lockdown, we used an R_0_ of 1.2 equal to an effectiveness of 57%. The same effectiveness was used for the *uniform measures* scenario, as it is assumed that people cannot comply with a stricter set of measures. The effective R_t_, however, may be lower as it is reduced by the ImR.

Many countries opted for strict measures to control the COVID-19 epidemic and to prevent capacity problems in their healthcare systems. This resulted in a comparatively low ImR at the end of the previous wave. Although strict measures keep the number of infections and the number of deaths low, they call for the adoption of strict measures also in the next wave, resulting into continuative negative consequences for the economy, and for the social and mental health of the people. However, the simulation results obtained with *differential measures* scenarios show that a strategy that limits the number of deaths while increasing the ImR in an acceptable way is possible. The *differential measures A* scenario isolates the older than 60 share of the population and reduces the share of incremental deaths for this group compared with *uniform measures* or *no-measures* and, although the total number of deaths is higher than for *uniform measures*, it does bring the ImR to a higher level, which is beneficial for limiting the consequences of following waves. Opting for *uniform measures* would not only produce severe adverse effects on the economy but also affect the psychosocial and mental health of the population (35), and together reduce the quality of life. On the other hand, the *differential measures A* scenario would not put strict measures on the younger than 60 part of the population and keep them productive and socially active benefitting their mental health (36, 37). The stricter *differential measures B* scenario, which reduces the number of work contacts, showed fewer incremental deaths than *differential measures A*. The two *differential measures* scenarios give countries an option to shift between these two strategies, depending on the social and economic costs of the measures and on the current state of the epidemic. Differentiation of measures according to age and location offers policy makers the option to balance economic, social, and health benefits until an effective vaccination is available and applied sufficiently.

The two demographic dimensions that were used to characterize the two prototype countries, age composition, and social contact structure have a major impact on virus transmission and the resulting infections and deaths. As expected, the higher share of people older than 60 in prototype S led to more deaths than in prototype NW. It is noteworthy that although prototype S has only 14% more persons in that group compared with prototype NW, it led to 89 and 88% more deaths for the same *no-measures* scenarios after an effective and less effective previous lockdown, respectively. This shows that the underlying dynamics of the contact structure play a significant role for the infections among groups and the resulting deaths. This is also visible for *differential measures A*, which lead to 342% more incremental deaths for prototype S compared with NW, and shows the need for *differential measures B*, where more restricting measures are implemented in other age groups. Therefore, the age composition and contact structure should not be ignored when designing control measures, as seemingly small differences in > 60-year-old shares can lead to a dramatic increase in the potential death toll, requiring additional differential measures to be enacted.

Our study demonstrates that the effectiveness of the previous lockdown has a significant influence on the severity of the next wave in terms of numbers of infections and deaths. A high ImR at the beginning of the next wave comes at the expense of more deaths depending on the scenario but limits the number of incremental infections and deaths. The results show that for the NW prototype, the cumulative number of deaths (all waves) is 33,138 with a low initial ImR (0.14) at the start of the next wave after an effective previous lockdown, whereas the cumulative number of deaths was 20,538 deaths for a high initial ImR (0.34) at the start of the next wave after a less effective previous lockdown. Although it can be argued that less stringent lockdown measures could lead to the collapse of the healthcare system, it can also be interpreted as finding a balance between healthcare capacity and social and economic requirements. In practice, this would mean that instead of reducing the number of daily cases as quickly as possible, a certain number of socially and economically acceptable cases are maintained and spreading the pressure on the health system over time, so that it can be handled better while limiting economic losses. Therefore, when designing the lockdown measures, it is not only recommended to look at the infections and deaths of next wave but also to the impact of these same lockdown measures in the following waves. This coincides with the worries expressed by Anderson et al. who stated that a low vaccination level, in this study, a low ImR, and SARS-CoV-2 will become endemic (38).

This study has several limitations. The first is that we did not include the number of available hospital and ICU beds in our study. Although countries tended to adjust their lockdown measures to the available capacity, the health system capacity would not change the conclusions as the impact of the simulated *differential measures* is well-below that of the *uniform measures* and thus within the existing policies. That said, including them would likely increase the number of infections and thus deaths for “no-measures” scenario and in maybe for the “uniform” scenario. A second limitation is that we assumed that the virus would spread homogenously throughout the population, while in the reality, transmission occurs mainly within clusters where local characteristics, time, and distance play an important role (39). A model based on cluster-wise transmission would be complex and difficult due to the need of detailed data. Nonetheless, the conclusions are still applicable if policy makers use the recommendations of this study to geographical clusters in their countries and adjust the policy upon those. Future research on the specific characteristics on the transmission can help to increase the validity of the model. Third, we only simulated two prototype countries with only two dimensions. Any other demographical characteristic was left out, as it was assumed that age was the most important differentiating variable with available data (40). As the goal of the study was to compare two protype countries in relation to specific scenarios, future studies could elaborate on this concerning more dimensions, such as immunity duration, vaccine efficacy and secondary effects, sequelae, ethnic characteristics, and corresponding sensitivity analysis. The fourth limitation is that we assumed persons would not be reinfected with COVID-19. Although there are cases of reinfection, they are rare and much less severe. A study done in Qatar confirmed this assumption and estimated the risk of reinfection at 0.01% (95% CI: 0.01–0.02%) with no deaths (41). Another study is in line with this and confirms that SARS-CoV-2-specific T-cell immunity is maintained at 6 months following primary infection (42, 43). Although immunity duration studies are still in progress, reduced duration could affect the outcomes of this study. Specifically, less effective previous waves would be affected in a next wave as reinfection would be more likely and increase the need for measures, likely differential measures. The fifth limitation is that we only considered contact reduction by limiting access to certain locations and age groups. However, other interventions could be implemented to reach similar results, such as face masks in the school place. If a similar reduction is reached, results of our analysis will then be the same. Finally, our study was retrospective, so we did not consider the quick introduction of effective COVID-19 vaccination as currently underway. At the moment of writing of this paper, many countries have already advanced vaccination campaigns. However, according to the current vaccination rates of some European countries and other counties in the world, it can be reasonably assumed that herd immunity will not be reached in the next few months, and we might be prepared to face other waves, for which the conclusions of this study are still relevant.

Notably, the lessons learned from our retrospective analysis are 3-fold. First, the proposed SIRD model is a powerful and flexible tool, which can be calibrated on various (prototype) countries, and seems ready-for-use to incorporate vaccination strategies. Second, the effectiveness of interventions, likely including vaccination, highly depends on the number of previous infections or immune citizens in the population at the start of the intervention, which is thus a key quantity that should be estimated and considered when planning intervention measures. Third, the effect of the number of people older than 60 should not be underestimated. Altogether, these lessons can serve policy makers when defining and designing measures fitting their situation to contain the COVID-19 epidemic. Depending on the adverse economic and social impact of lockdown, differential measures can offer help.

## Conclusions

This retrospective study shows that for controlling the COVID-19 epidemic in an acceptable way and for a longer period, differential measures are a worthwhile option. Such measures can keep the productive part of the economy active, protect the high-risk population, increase the immunity level, with a limited number of deaths, and simplify possible next waves. Differential measures where the older than 60 part of the population is isolated may keep the <60-year-old individuals productive and active, which reduce economic, social, and mental problems.

Next, results do change per prototype country and show that the age composition and contact structure are relevant for the design of control measures. A higher share of older than 60 in the population causes significantly more than proportional deaths but also reduces the effectiveness of differential measures where the older than 60 group is isolated such that additional contact-reducing measures will be needed in other age groups. Age differences should therefore be considered when designing measures. Indeed, they reflect a core component of the dynamic SIRD model.

Finally, this study shows that a vision beyond the current wave is a key to effectively design control measures, and that the age composition and contact structure of a country are critical aspects of the measures' evaluation. For the assessment and planning non-pharmacological interventions and vaccination campaigns, the effectiveness of previous interventions and size of previous waves should be considered, in addition to the specific nature of the intervention, and its impact on protecting against infection, disease, and transmission. Therefore, policy makers would need to adjust the measures to the demographic situation and the severity of previous waves. This means that at least similar measures would be required for a next wave if a previous wave was very effective. Additionally, an older population would require more strict isolation measures compared with other younger populations.

## Data Availability Statement

The raw data supporting the conclusions of this article will be made available by the authors, without undue reservation.

## Author Contributions

AH and KC-P contributed in conceptualization, performed formal analysis, and conducted the investigation. AH, KC-P, and LZ involved in methodology. AH provided the software and involved in project administration. JS, MP, LZ, and JW contributed in validation. MP and JS provided the resources. AH, KC-P, and JS wrote the original draft. JS, MP, and JW contributed in writing, reviewing, and editing. MP supervised the data. All authors have read and agreed to the published version of the manuscript.

## Conflict of Interest

The authors declare that the research was conducted in the absence of any commercial or financial relationships that could be construed as a potential conflict of interest. The reviewer RF declared a shared affiliation, with no collaboration, with the authors, to the handling editor at the time of the review.

## Publisher's Note

All claims expressed in this article are solely those of the authors and do not necessarily represent those of their affiliated organizations, or those of the publisher, the editors and the reviewers. Any product that may be evaluated in this article, or claim that may be made by its manufacturer, is not guaranteed or endorsed by the publisher.

## Footnotes

^1^For example, the country-wide measures in the Netherlands started March 9^th^, in Italy country-wide measures started on March 8^th^. In both countries, measures, although reduced, were still in place on 1 July 1^st^.
