# Supplementary material for: Reconstructing the Effectiveness of Policy Measures to Avoid Next-Wave COVID-19 Infections and Deaths Using a Dynamic Simulation Model: Implications for Health Technology Assessment
Source: Front Med Technol. 2022 Jan 26;3:666581. doi: 10.3389/fmedt.2021.666581 (PMC8825500; doi:10.3389/fmedt.2021.666581)
Supplement: Supplementary file 1 [file Data_Sheet_1.docx]

Supplementary Material

# Appendix A. Contact matrices

**Supplementary Figure 1.** The contact matrices used for the simulations.

# Appendix B. Differential equations

$$\frac{dSi}{dt}=-\beta S_{i}\sum_{j} C_{ij}I_{j}/N_{j}$$

$$\frac{dI_{i}}{dt}=\beta S_{i}\sum_{j} C_{ij}I_{j}/N_{j}-\gamma I_{i}$$

$$\frac{dR_{i}}{dt}=f_{i}\gamma I_{i}$$

$$\frac{dD_{i}}{dt}=(1-f_{i})\gamma(I_{i})$$

Where: $f_{j}=recovery rate in group j$

S=Susceptible, I=Infectious, R=Recovered, D=Death

C = Contact matrix adjusted for non-reciprocity

N_j_ = total population in group j

$\beta=R_{0}\left( \gamma\right)/(MaxEigenvalue C)$

$$R_{0}=Basic reproduction number$$

$$\gamma=\frac{1}{Infectious days}$$

# Appendix C. Case-fatality rate estimation

Supplementary Table 1. Case-fatality rate estimation

| **Age group** | **confirmed deaths** | **confirmed cases** | **Case- fatality rate** | **corrected cases** | **cases model** | **Case-fatality rate model** |
| --- | --- | --- | --- | --- | --- | --- |
| **0-19** | 1 | 1230 | 0.1% | 34,052 | 431,846 | 0.00023% |
| **20-39** | 14 | 10178 | 0.1% | 281,776 | 487,249 | 0.00287% |
| **40-59** | 177 | 15271 | 1.2% | 422,775 | 440,828 | 0.04015% |
| **≥60** | 5940 | 24011 | 24.7% | 664,740 | 77,147 | 7.69957% |
| **Total** | 6132 | 50690 | 12.1% | 1,403,342 | 1,437,070 | 0.42670% |
|  |  |  |  |  |  |  |
| confirmed deaths: number of deaths on July 7^th^, 2020 in the Netherlands | | | | | |  |
| confirmed cases: number of cases on July 7^th^, 2020 in the Netherlands | | | | | |  |
| CFR: case-fatality rate according to the confirmed deaths and cases | | | | | | |
| Corrected cases: number of cases corrected using antibody positive cases study of May 18^th^ 2020 | | | | | | |
| cases model: number of cases according to the SIR model on comparable day | | | | | |  |
|  | (model based on startday 51, R_0_=2.8, PE=0.75, infectious period=7) | | | | | |
| CFR model: case-fatality rate using the confirmed deaths and the cases of the model | | | | | | |

# Appendix D. Weekly new infections and deaths in different scenarios

**Supplementary Figure 2**. Weekly new infections and deaths for the eight scenarios for country NW and S. Starting at the first week of the epidemic including first-wave and next-wave simulation results.

# Appendix E. Reproduction rate (Rt) global distribution


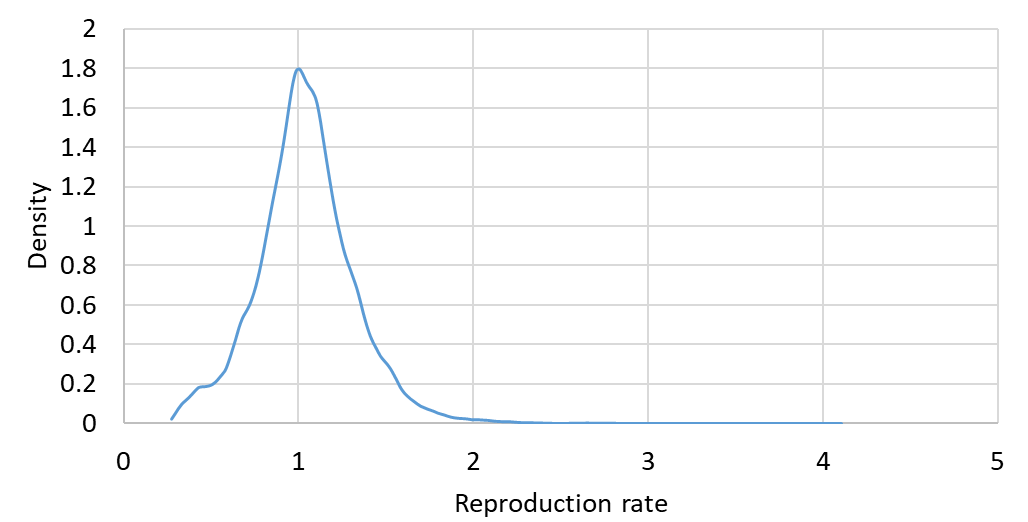


Figure 3. Distribution of the COVID-19 Reproduction rate between 1-4-2020 and 1-4-2021. Approximately 1% of the daily reported numbers of 215 countries is above a reproduction rate 1.8.
